# Supplementary material for: Integrated information theory (IIT) 4.0: Formulating the properties of phenomenal existence in physical terms
Source: PLoS Comput Biol. 2023 Oct 17;19(10):e1011465. doi: 10.1371/journal.pcbi.1011465 (PMC10581496; doi:10.1371/journal.pcbi.1011465)
Supplement: S1 Text — Operational process for resolving ties due to maxima / minima in the IIT algorithm. (PDF) [file pcbi.1011465.s001.pdf]

Integrated information theory (IIT) 4.0: Formulating the properties of phenomenal existence in physical terms

Larissa Albantakis[1¶], Leonardo Barbosa[1,2¶], Graham Findlay[1,3¶], Matteo Grasso[1¶], Andrew M Haun[1¶], William Marshall[1,4¶], William GP Mayner[1,3¶], Alireza Zaeemzadeh[1¶], Melanie Boly[1,5], Bjørn E Juel[1,6], Shuntaro Sasai[1,7], Keiko Fujii[1], Isaac David[1], Jeremiah Hendren[1,8], Jonathan P Lang[1], Giulio Tononi[1*]

1 Department of Psychiatry, University of Wisconsin, Madison, Wisconsin, USA

2 Fralin Biomedical Research Institute at VTC, Virginia Tech, Roanoke, Virgina, USA

3 Neuroscience Training Program, University of Wisconsin, Madison, Wisconsin, USA

4 Department of Mathematics and Statistics, Brock University, St. Catharines, Ontario, Canada

5 Department of Neurology, University of Wisconsin, Madison, Wisconsin, USA

6 Institute of Basic Medical Sciences, University of Oslo, Oslo, Norway

7 Araya Inc., Tokyo, Japan

8 Graduate School Language & Literature, Ludwig Maximilian University of Munich, Munich, Germany

¶These authors contributed equally to this work.

* gtononi@wisc.edu

S1 - Resolving ties in the IIT algorithm

The information postulate requires that a system's cause–effect power is specific: the system in its current state must select a specific cause–effect state for its units. Likewise, mechanisms within the system must select a specific cause and effect state over their purviews. The exclusion postulate requires that a complex must be constituted of a definite set of units, and that mechanisms within the complex specify a definite cause and effect. By the principle of maximal existence, cause–effect states,

complexes, and the cause–effect states of a mechanism within the system are identified as those with maximal cause–effect power.

However, for systems with built-in symmetries in their architecture or in the input–output functions of their units, multiple sets of units or cause–effect states may "tie" for maximal intrinsic information or integrated information [1–4]. While such ties are frequently encountered in small, deterministic toy models, they are unlikely to occur in realistic systems. In fact, IIT requires a degree of indeterminism at the micro-level from first principles. Ties are a consequence of symmetries in the substrate TPMs, which also become increasingly unlikely for larger, more realistic systems and are thus unlikely to play any role in the empirical application and evaluation of IIT. Nevertheless, such ties should be resolved in line with IIT's postulates and principles, as we outline in the following for algorithmic purposes. In general, ties that occur at an intermediate step in the algorithm are resolved based on the principle of maximal existence by considering the subsequent postulates (essential requirements for existence) in order.

Maximal substrates can be identified using an iterative algorithm (25). A maximal substrate excludes all overlapping systems with lower φ_s from existing as complexes. If overlapping systems tie for $\max\limits_{S \subseteq U_k} \varphi_s(\mathcal{T}_e, \mathcal{T}_c, s)$, we apply the maximum existence principle taking their respective Φ values (composition) into consideration and choose the system with maximal structure integrated information Φ as the complex. In the rare case that two or more such systems also tie in Φ, these systems do not comply with the exclusion postulate. For this reason, they do not qualify as complexes and we choose the next best system (based on φ_s) that is unique [5] (see also [2] for a similar line of reasoning).

For a system, the maximal cause–effect state $s' = \{s'_c, s'_e\}$ is the one that maximizes the system's intrinsic cause and effect information. However, if multiple states comply with equation (12), we select the one for which the system specifies the maximal integrated information $\varphi_s(\mathcal{T}_e, \mathcal{T}_c, s, \theta')$ (21) over its minimum partition θ'. This is again in line with the principle of maximal existence: being tied for intrinsic information, it is the integrated information φ_s that determines in which cause-effect state the system "exists the most."

Remaining ties in which multiple cause-effect states specify the same φ_s rarely matter for system selection, but need to be resolved in order to determine the system's cause–effect structure. By the maximum existence principle, we choose the cause–effect

state that maximizes the system's structure integrated information Φ. As above, in the rare case that two or more states also tie in Φ, the system does not comply with the information postulate and thus does not qualify as a complex (unless the cause-effect structures are actually identical from the intrinsic perspective, in which case the tie would be extrinsic and not a violation of the information postulate).

The cause or effect state of a mechanism within the system for a candidate purview is first selected based on its intrinsic information $\text{ii}(m, z)$ (36). Next, we compare the integrated information $\varphi(m, Z)$ (42) of all maximal cause or effect states across all possible purviews (including all possible ties in $\text{ii}(m, z)$ within a candidate purview) to identify the maximally irreducible cause or effect $z^*_{c/e}$ of the mechanism within the system (45). By the maximum existence postulate, potential ties in $\max \varphi_d(m, Z)$ and thus in the cause–effect state $z^*_{c/e}$ of a distinction may be resolved at the level of the cause–effect structure, by selecting the $z^*_{c/e}$ that maximizes the system's structure integrated information Φ. Accordingly, in case of state ties within the same purview, we select the state that is congruent with the system's cause–effect state s'. In case of ties across different purviews, the maximal cause–effect state will generally correspond to the one that supports the most relations with other distinctions, which typically favors larger purviews.

References

1. Krohn S, Ostwald D. Computing integrated information. Neuroscience of Consciousness. 2017;2017(1). doi:10.1093/nc/nix017.

2. Moon K. Exclusion and Underdetermined Qualia. Entropy. 2019;21(4):405. doi:10.3390/e21040405.

3. Hanson JR, Walker SI. On the Non-uniqueness Problem in Integrated Information Theory. 2020;doi:10.1101/2021.04.07.438793.

4. Barbosa LS, Marshall W, Albantakis L, Tononi G. Mechanism Integrated Information. Entropy. 2021;23(3):362.

5. Marshall W, Grasso M, Mayner WG, Zaeemzadeh A, Barbosa LS, Chastain E, et al. System Integrated Information. Entropy. 2023;25.
