# Supplementary material for: Integrated information theory (IIT) 4.0: Formulating the properties of phenomenal existence in physical terms
Source: PLoS Comput Biol. 2023 Oct 17;19(10):e1011465. doi: 10.1371/journal.pcbi.1011465 (PMC10581496; doi:10.1371/journal.pcbi.1011465)
Supplement: S2 Text — Summary of the changes in IIT 4.0 relative to earlier versions of the theory. (PDF) [file pcbi.1011465.s002.pdf]

Integrated information theory (IIT) 4.0: Formulating the properties of phenomenal existence in physical terms

Larissa Albantakis[1¶], Leonardo Barbosa[1,2¶], Graham Findlay[1,3¶], Matteo Grasso[1¶], Andrew M Haun[1¶], William Marshall[1,4¶], William GP Mayner[1,3¶], Alireza Zaeemzadeh[1¶], Melanie Boly[1,5], Bjørn E Juel[1,6], Shuntaro Sasai[1,7], Keiko Fujii[1], Isaac David[1], Jeremiah Hendren[1,8], Jonathan P Lang[1], Giulio Tononi[1*]

1 Department of Psychiatry, University of Wisconsin, Madison, Wisconsin, USA

2 Fralin Biomedical Research Institute at VTC, Virginia Tech, Roanoke, Virginia, USA

3 Neuroscience Training Program, University of Wisconsin, Madison, Wisconsin, USA

4 Department of Mathematics and Statistics, Brock University, St. Catharines, Ontario, Canada

5 Department of Neurology, University of Wisconsin, Madison, Wisconsin, USA

6 Institute of Basic Medical Sciences, University of Oslo, Oslo, Norway

7 Araya Inc., Tokyo, Japan

8 Graduate School Language & Literature, Ludwig Maximilian University of Munich, Munich, Germany

¶These authors contributed equally to this work.

* gtononi@wisc.edu

S2 - Comparison to IIT 1.0–3.0 and subsequent publications

As highlighted in the main text, IIT is a work in progress. While the core theory has remained the same, its formal framework has been progressively refined and extended [1–4]. Compared to prior versions (IIT 1.0 [1, 2], IIT 2.0 [3, 5, 6], and IIT 3.0 [4, 7–9]), IIT 4.0 presents a more complete, self-consistent formulation. The most

notable advances in IIT 4.0 include the introduction of an Intrinsic Difference (ID) measure [10, 11] that is uniquely consistent with IIT's postulates, the explicit assessment of causal relations [12], and a more exact translation of the axioms into postulates. Because IIT 3.0 already included a comparison to IIT 1.0 and 2.0 (see [4], Supporting Information Text S1), we mainly focus on subsequent developments.

Axioms and postulates

The starting point of IIT has always been phenomenology, but the axioms and postulates of the theory were first explicitly presented in IIT 3.0 [4, 13]. The updated 4.0 exposition of IIT's axioms explicitly separates phenomenal existence, which is not a property, from intrinsicality, which is one of the essential properties of phenomenal existence. Accordingly, the existence of experience is introduced as IIT's foundational, zeroth axiom. The remaining five axioms (intrinsicality, information, integration, exclusion, and composition) capture the essential properties that are immediate and irrefutably true of every conceivable experience.

Compared to IIT 3.0, the formulation of the axioms has been refined to avoid misunderstandings [14, 15] and to highlight their immediacy and irrefutability. The formulation of IIT's postulates has been updated accordingly with the objective of tracking the phenomenal axioms as closely as possible. For example, conforming more closely to the information axiom, the information postulate requires that the system must select a specific cause–effect state over its units. The composition axiom now highlights both phenomenal distinctions and their relations. By the composition postulate, phenomenal distinctions and relations are accounted for in physical terms by causal distinctions and relations. Because only an experience that exists intrinsically, in a way that is specific, irreducible, and definite can also be structured, composition takes the final position in the ordering of the axioms and postulates.

Background Conditions

The IIT 4.0 mathematical framework updates the treatment of background conditions (units $W = U \setminus S$). In IIT 3.0 [4], the background units were fixed (conditioned) in their current state for the evaluation of effects, and fixed (conditioned) in their actual past

state for the evaluation of causes. In publications since then, the actual past state of background units was considered to be unavailable from the intrinsic perspective of the system, so instead the background units were fixed (conditioned) in their current state for evaluating both causes and effects [11, 12, 16, 17]. However, fixing the background conditions in the current state for evaluating causes leads to situations where the current state is unreachable (no cause). In IIT 4.0, the treatment of background conditions is updated to causally marginalize the background units, conditional on the current state of the universe (see Identifying substrates of consciousness). This formulation avoids the problem of unreachable states, while also only requiring knowledge of the current state of background units (the 'context' for the causal powers analysis).

Identifying maximal substrates

The IIT 4.0 formalism to identify maximal substrates was first described in detail in [17]. Maximal substrates, or complexes, are identified based on their system integrated information φ_s (as in IIT 2.0 but unlike IIT 3.0, which evaluated integration after composition). System partitions remain directional (as in IIT 3.0). In IIT 4.0, the minimum partition (MIP) is identified as the partition with minimal integrated information (φ_s), normalized by the maximal possible value of φ_s across this partition for an arbitrary TPM of the same dimensions (23). In this way, the MIP is sensitive to the fault lines of a candidate system, rather than defaulting to partitions of individual system units. The IIT 4.0 analysis is state-dependent (as in IIT 2.0 and 3.0) and requires positive cause and effect power for a system to exist (as in IIT 1.0 and 3.0).

Measuring intrinsic information

Supplanting prior measures such as the Kullback-Leibler divergence (KLD, IIT 2.0 [3]), or the (extended) Earth Mover's Distance (EMD, IIT 3.0, [4]), the IIT 4.0 formalism features a newly developed Intrinsic Difference (ID) measure [10], which uniquely complies with the postulates of IIT. Formally:

Theorem 1 *Let* $(p, q) \in \mathcal{T}_U$ *be two probability distributions, and* $D\colon (\mathcal{T}_U, \mathcal{T}_U) \to \mathbb{R}$*,*

where D satisfies Properties I, II and III defined in [10]. Then

$$D(p,q) = \max_{u \in \Omega_U} \{f(p(u), q(u))\},$$

where

$$f(p(u), q(u)) = k\, p(u) \log \left(\frac{p(u)}{q(u)} \right), k \in \mathbb{R}^+. \tag{1}$$

The proof of the Theorem can be found in [10]. Note that ID is related to the KLD, which can be viewed as an average of the point-wise mutual information across states and is an additive measure. By contrast, the ID is defined based on the state that maximizes the difference between distributions (specificity property). Accordingly, the intrinsic information specified by a system (or mechanism) over a cause or effect state is evaluated as a product of informativeness and selectivity, which makes it subadditive if $p < 1$, that is, if cause–effect power is spread over more than one state. As intrinsic information is evaluated over specific cause or effect states, its maximal value over a state distribution identifies the specific cause or effect state selected by the system (or mechanism), in line with the information postulate. The intrinsic effect information ii_e is equivalent to the ID between the constrained and unconstrained effect probability distributions, but the intrinsic cause information ii_c is not because of the use of backwards cause probabilities for selectivity (see main text).

Causal distinctions

Distinctions capture how the various system subsets specify system subsets as their cause and effect within the system. In IIT 3.0, distinctions were called "concepts" (which composed to a "conceptual" structure), a term that could generate unnecessary misunderstandings [12]. An updated formalism to identify causal distinctions was first presented in [11]. As in IIT 3.0, causes and effects must be specified in a way that is irreducible ($\varphi_d > 0$). Unlike IIT 3.0, in IIT 4.0 distinctions select a specific state as a cause and an effect. The definition of cause and effect probabilities $\pi(z \mid m)$ ((30), (33) and (29)) remains unchanged, but is now presented more formally in terms of product probabilities, rather than referring to "virtual elements" [18, 19]). In IIT 4.0, a mechanism selects a specific cause and effect state based on the Intrinsic Difference (ID)

measure introduced in [10] (see above). The set of permissible partitions $\theta \in \Theta(M, Z)$ (38) has also been updated [11, 19] to ensure that partitions are always "disintegrating" the mechanism.

The present formulation includes several updates compared to [11]. First, the cause–effect state z' is selected based on the intrinsic information $\text{ii}_e(m, Z)$ (36), before evaluating its integrated information $\varphi(m, Z)$ (42) for z'. This is because the cause and effect of m (its cause-effect state) should be determined by the mechanism as a whole, independent of how it can be partitioned. Second, we evaluate intrinsic information without the absolute value, as in [10], because, to comply with the existence postulate, a mechanism's cause state should be one that would increase the probability of its current state, and its effect state one whose probability would be increased by the mechanism being in its current state. Third, to correctly capture this increase in probability on the cause side, the informativeness term is expressed in terms of forward probabilities (as opposed to backward probabilities employing Bayes rule) also on the cause side for ii_c and φ_c, evaluating the increase in probability of the current state due to the cause state. Fourth, we have updated the resolution of ties at the level of distinctions according to the principle of maximal existence (see S1 Text). Finally, a candidate distinction only contributes to the system's cause–effect structure if its maximal cause–effect state z^* is congruent with the maximal cause–effect state s' of the system.

Causal Relations

Relations bind together a set of causal distinctions over a congruent overlap between their causes and/or effects. Developing an explicit account of phenomenal relations in terms of causal relations was a main goal of IIT since IIT 1.0. IIT 3.0 employed a distance metric—the Earth Mover's Distance—that was sensitive to whether different distinctions ("concepts") had similar cause–effect repertoires, but relations did not figure explicitly in the formalism despite their central role in characterizing experience. An explicit account was first described in [12]. The IIT 4.0 formalism further distinguishes between relations (which bind a set of distinctions with overlapping causes and/or effects) and the faces of a relation (which specify the maximal overlap of a set of purviews and jointly characterize the type of the relation). Moreover, the amount of

information specified by a distinction over the overlap and the way relation partitions are assessed differs from the original account [12]. Because distinctions are irreducible components within the cause–effect structure upon which relations are built, a distinction involved in a relation contributes its entire φ_d, weighted by the extent of its joint overlap (55). For this reason, we do not recompute the irreducible information of the mechanism m of a distinction $d(m, z^*, \varphi_d)$ over the candidate overlap o. In [12], distinctions contributing to a relation were partitioned by "noising" the interactions among distinction units.

Φ-structures

In IIT 4.0, a system is a substrate of consciousness (a complex) if it corresponds to a maximum of system integrated information φ_s, as determined through information, integration, and exclusion. This is similar to IIT 2.0 [3], although in that case only causes (and not effects) were evaluated. The quality of the experience is identical to the Φ-structure of distinctions and relations unfolded from the complex. The quantity of experience corresponds to the Φ value, the sum of the integrated information of the distinctions and relations that compose the Φ-structure. In IIT 3.0, the determination of the complex through information, integration, and exclusion took into account its compositional structure, although without explicit relations. However, the Φ value corresponding to the quantity of consciousness only captured the distinctions affected by the minimum partition, as opposed to all the distinctions (and relations) unfolded from a maximally irreducible substrate. Because Φ is not evaluated with respect to a MIP, there is no normalization involved in determining Φ (in contrast to φ_S). Instead, Φ captures the complete structure integrated information of a complex in the form of a sum over the integrated information φ of all components of the complex (its distinctions and relations), where the sum was chosen as the simplest option that captures all that exists within the complex. As such, Φ in IIT 4.0 is more aligned with the common-sense notion that the quantity of consciousness relates to the "richness" or "vividness" of an experience considering all its contents. However, whether a system exists as one integrated entity is evaluated by its system integrated information φ_s, which is not compositional.

References

1. Tononi G, Sporns O. Measuring information integration. BMC neuroscience. 2003;4(31):1–20.

2. Tononi G. An information integration theory of consciousness. BMC neuroscience. 2004;5:42. doi:10.1186/1471-2202-5-42.

3. Balduzzi D, Tononi G. Integrated information in discrete dynamical systems: motivation and theoretical framework. PLoS Comput Biol. 2008;4(6):e1000091. doi:10.1371/journal.pcbi.1000091.

4. Oizumi M, Albantakis L, Tononi G. From the Phenomenology to the Mechanisms of Consciousness: Integrated Information Theory 3.0. PLoS Computational Biology. 2014;10(5):e1003588. doi:10.1371/journal.pcbi.1003588.

5. Balduzzi D, Tononi G. Qualia: the geometry of integrated information. PLoS computational biology. 2009;5(8):e1000462. doi:10.1371/journal.pcbi.1000462.

6. Tononi G. Consciousness as integrated information: a provisional manifesto. Biol Bull. 2008;215(3):216–242. doi:215/3/216 [pii].

7. Tononi G. Integrated Information Theory of Consciousness: An Updated Account. Arch Ital Biol. 2012;150:56–90.

8. Mayner WGP, Marshall W, Albantakis L, Findlay G, Marchman R, Tononi G. PyPhi: A toolbox for integrated information theory. PLoS Computational Biology. 2018;14(7):e1006343. doi:10.1371/journal.pcbi.1006343.

9. Kleiner J, Tull S. The Mathematical Structure of Integrated Information Theory. Frontiers in Applied Mathematics and Statistics. 2021;6:74. doi:10.3389/FAMS.2020.602973/BIBTEX.

10. Barbosa LS, Marshall W, Streipert S, Albantakis L, Tononi G. A measure for intrinsic information. Scientific Reports. 2020;10(1):18803. doi:10.1038/s41598-020-75943-4.

11. Barbosa LS, Marshall W, Albantakis L, Tononi G. Mechanism Integrated Information. Entropy. 2021;23(3):362.

12. Haun AM, Tononi G. Why Does Space Feel the Way it Does? Towards a Principled Account of Spatial Experience. Entropy. 2019;21(12):1160. doi:10.3390/e21121160.

13. Tononi G, Boly M, Massimini M, Koch C. Integrated information theory: from consciousness to its physical substrate. Nature Reviews Neuroscience. 2016;17(7):450–461. doi:10.1038/nrn.2016.44.

14. Bayne T. On the axiomatic foundations of the integrated information theory of consciousness. Neuroscience of Consciousness. 2018;2018(1). doi:10.1093/nc/niy007.

15. Merker B, Williford K, Rudrauf D. The Integrated Information Theory of consciousness: A case of mistaken identity. Behavioral and Brain Sciences. 2021;21:1–72. doi:10.1017/S0140525X21000881.

16. Hoel EP, Albantakis L, Marshall W, Tononi G. Can the macro beat the micro? Integrated information across spatiotemporal scales. Neuroscience of Consciousness. 2016;2016(1).

17. Marshall W, Grasso M, Mayner WG, Zaeemzadeh A, Barbosa LS, Chastain E, et al. System Integrated Information. Entropy. 2023;25.

18. Krohn S, Ostwald D. Computing integrated information. Neuroscience of Consciousness. 2017;2017(1). doi:10.1093/nc/nix017.

19. Albantakis L, Marshall W, Hoel E, Tononi G. What caused what? A quantitative account of actual causation using dynamical causal networks. Entropy. 2019;21(5):459. doi:10.3390/e21050459.
