# Supplementary material for: Integrated information theory (IIT) 4.0: Formulating the properties of phenomenal existence in physical terms
Source: PLoS Comput Biol. 2023 Oct 17;19(10):e1011465. doi: 10.1371/journal.pcbi.1011465 (PMC10581496; doi:10.1371/journal.pcbi.1011465)
Supplement: S3 Text — Statement and proof of theorems describing the number of relations and the sum of their integrated information, ∑φr. (PDF) [file pcbi.1011465.s003.pdf]

Integrated information theory (IIT) 4.0: Formulating the properties of phenomenal existence in physical terms

Larissa Albantakis[1¶], Leonardo Barbosa[1,2¶], Graham Findlay[1,3¶], Matteo Grasso[1¶], Andrew M Haun[1¶], William Marshall[1,4¶], William GP Mayner[1,3¶], Alireza Zaeemzadeh[1¶], Melanie Boly[1,5], Bjørn E Juel[1,6], Shuntaro Sasai[1,7], Keiko Fujii[1], Isaac David[1], Jeremiah Hendren[1,8], Jonathan P Lang[1], Giulio Tononi[1*]

1 Department of Psychiatry, University of Wisconsin, Madison, Wisconsin, USA

2 Fralin Biomedical Research Institute at VTC, Virginia Tech, Roanoke, Virginia, USA

3 Neuroscience Training Program, University of Wisconsin, Madison, Wisconsin, USA

4 Department of Mathematics and Statistics, Brock University, St. Catharines, Ontario, Canada

5 Department of Neurology, University of Wisconsin, Madison, Wisconsin, USA

6 Institute of Basic Medical Sciences, University of Oslo, Oslo, Norway

7 Araya Inc., Tokyo, Japan

8 Graduate School Language & Literature, Ludwig Maximilian University of Munich, Munich, Germany

¶These authors contributed equally to this work.

* gtononi@wisc.edu

S3 - Analytical solution for $\sum \varphi_r$ and the number of causal relations

Here, we show how the sum of the relation integrated information over all the causal relations ($\sum \varphi_r$) and the number of relations can be computed without assessing the relations individually. We only need the set of causal distinctions:

$$D(\mathcal{T}_e, \mathcal{T}_c, s) = \{d(m) \;:\; m \subseteq s,\; \varphi_d(m) > 0,\; z_c^*(m) \subseteq s'_c,\; z_e^*(m) \subseteq s'_e\},$$

where $d(m) = (m, z^*(m), \varphi_d(m))$ and $z^*(m) = \{z_c^*(m), z_e^*(m)\}$.

Analytical computation of $\sum \varphi_r$

Given a subset of distinctions $\boldsymbol{d} \subseteq D(\mathcal{T}_e, \mathcal{T}_c, s)$ with $|\boldsymbol{d}| \geq 2$, any subset $\boldsymbol{z}$ of purviews that contains either the cause, or effect, or both the cause and effect of each distinction $d \in \boldsymbol{d}$ and overlap congruently defines a relation face f with face overlap $o_f^* = \bigcap_{z \in \boldsymbol{z}} z$. The relation overlap is further defined as the union of the face overlaps $\bigcup_{f \in \boldsymbol{f}(\boldsymbol{d})} o_f^*$, where $\boldsymbol{f}(\boldsymbol{d})$ represents the set of all the faces over the distinction set $\boldsymbol{d}$. Here, intersection and union take into account both the units and their states.

First, we can show:

$$\bigcup_{f \in \boldsymbol{f}(\boldsymbol{d})} o_f^* = \bigcap_{d \in \boldsymbol{d}} \left(z_c^*(d) \cup z_e^*(d)\right),$$

by proving any unit n in $\bigcup_{f \in \boldsymbol{f}(\boldsymbol{d})} o_f^*$ is in $\bigcap_{d \in \boldsymbol{d}} \left(z_c^*(d) \cup z_e^*(d)\right)$ and vice versa:

$$n \in \bigcup_{f \in \boldsymbol{f}(\boldsymbol{d})} o_f^* \iff \exists f \in \boldsymbol{f}(\boldsymbol{d}), n \in o_f^* \iff \forall d \in \boldsymbol{d}, n \in z_c^*(d) \text{ or } n \in z_e^*(d)$$
$$\iff \forall d \in \boldsymbol{d}, n \in z_c^*(d) \cup z_e^*(d) \iff n \in \bigcap_{d \in \boldsymbol{d}} \left(z_c^*(d) \cup z_e^*(d)\right)$$

This helps us to rewrite the relation integrated information of a set of distinctions $\boldsymbol{d} \subseteq D(\mathcal{T}_e, \mathcal{T}_c, s)$ with $|\boldsymbol{d}| \geq 2$ as:

$$\left|\bigcap_{d \in \boldsymbol{d}} \left(z_c^*(d) \cup z_e^*(d)\right)\right| \min_{(z_d, \varphi_d) \in \boldsymbol{d}} \frac{\varphi_d}{|z_c^*(d) \cup z_e^*(d)|}.$$

We further define the set of $z_c^*(d) \cup z_e^*(d)$ of all distinctions in D and their corresponding distinction integrated information as:

$$\mathcal{Z}(\mathcal{T}_e, \mathcal{T}_c, s) = \left\{\left(z_c^*(m) \cup z_e^*(m), \varphi(m)\right) : (m, z^*(m), \varphi_d(m)) \in D(\mathcal{T}_e, \mathcal{T}_c, s)\right\}.$$

Now, given a single node n in a specific state, we can find all the distinctions that contain n in that state in their cause, or effect, or both purviews as:

$$\mathcal{Z}(n) = \{(z, \varphi) : (z, \varphi) \in \mathcal{Z}(\mathcal{T}_e, \mathcal{T}_c, s), n \in z\}. \tag{1}$$

Any subset of $\mathcal{Z}(n)$ of size 2 or larger defines a relation whose overlap contains at least n. Formally, for $\boldsymbol{r} \subseteq \mathcal{Z}(n)$, $|\boldsymbol{r}| \geq 2$, there exists a relation with relation purview $\bigcap_{(z_d,\varphi_d)\in\boldsymbol{r}} z_d$ and integrated information value of:

$$\left|\bigcap_{(z_d,\varphi_d)\in\boldsymbol{r}} z_d\right| \min_{(z_d,\varphi_d)\in\boldsymbol{r}} \frac{\varphi_d}{|z_d|}.$$

Note that, by definition of $\mathcal{Z}(n)$ and $\mathcal{Z}(\mathcal{T}_e, \mathcal{T}_c, s)$, z_d is the union of cause and effect purviews. Using the definition of $\mathcal{Z}(\mathcal{T}_e, \mathcal{T}_c, s)$ and $\mathcal{Z}(n)$, we can write the sum of the integrated information of relations, except self-relations, as

$$\sum_{\substack{\boldsymbol{r}\subseteq\mathcal{Z}(\mathcal{T}_e,\mathcal{T}_c,s)\\ \boldsymbol{r}\geq 2}} \left|\bigcap_{(z_d,\varphi_d)\in\boldsymbol{r}} z_d\right| \min_{(z_d,\varphi_d)\in\boldsymbol{r}} \frac{\varphi_d}{|z_d|} = \sum_{n\in s'_c\cup s'_e} \sum_{\substack{\boldsymbol{r}\subseteq\mathcal{Z}(n)\\ |\boldsymbol{r}|\geq 2}} \min_{(z_d,\varphi_d)\in\boldsymbol{r}} \frac{\varphi_d}{|z_d|},$$

By factoring the sum over $\boldsymbol{r} \subseteq \mathcal{Z}(\mathcal{T}_e, \mathcal{T}_c, s)$ into two sums over the nodes n and the relations whose purview contains *at least* n, $\boldsymbol{r} \subseteq \mathcal{Z}(n), |\boldsymbol{r}| \geq 2$, we are overcounting each relation by a factor of its joint purview size $\left|\bigcap_{(z_d,\varphi_d)\in\boldsymbol{r}} z_d\right|$. For example, if a set of distinctions make up a relation $\boldsymbol{r}$ over two units n_1 and n_2, they all are members of both $\mathcal{Z}(n_1)$ and $\mathcal{Z}(n_2)$. Therefore, $\boldsymbol{r} \subseteq \mathcal{Z}(n_1)$ and $\boldsymbol{r} \subseteq \mathcal{Z}(n_2)$. This simplifies the summand to just $\min_{(z_d,\varphi_d)\in\boldsymbol{r}} \frac{\varphi_d}{|z_d|}$. To compute the inner sum, we can sort the distinctions in $\mathcal{Z}(n)$ by their $\frac{\varphi}{|z|}$ value in a non-decreasing order, such that $(z_{(1)}, \varphi_{(1)})$ has the summary est $\frac{\varphi}{|z|}$ ratio, $(z_{(2)}, \varphi_{(2)})$ has the second smallest $\frac{\varphi}{|z|}$ ratio, and so on. Then, we can compute the sum as:

$$\sum_{\substack{\boldsymbol{r}\subseteq\mathcal{Z}(n)\\ |\boldsymbol{r}|\geq 2}} \min_{(z_d,\varphi_d)\in\boldsymbol{r}} \frac{\varphi_d}{|z_d|} = \sum_{j=1}^{|\mathcal{Z}(n)|} \frac{\varphi_{(j)}}{|z_{(j)}|}(2^{|\mathcal{Z}(n)|-j} - 1).$$

In words, any subset $\boldsymbol{r} \subseteq \mathcal{Z}(n), |\boldsymbol{r}| \geq 2$, that contains $(z_{(1)}, \varphi_{(1)})$ will have $\min_{(z_d,\varphi_d)\in\boldsymbol{r}} \frac{\varphi_d}{|z_d|} = \frac{\varphi_{(1)}}{|z_{(1)}|}$. There are $2^{|\mathcal{Z}(n)|-1} - 1$ of such subsets. Similarly, there are $2^{|\mathcal{Z}(n)|-2} - 1$ subsets that contain $(z_{(2)}, \varphi_{(2)})$, but not $(z_{(1)}, \varphi_{(1)})$, etc. This helps us arrive at our final results:

$$\sum_{\substack{\boldsymbol{r}\subseteq\mathcal{Z}(\mathcal{T}_e,\mathcal{T}_c,s)\\|\boldsymbol{r}|\geq 2}} \left|\bigcap_{(z_d,\varphi_d)\in\boldsymbol{r}} z_d\right| \min_{(z_d,\varphi_d)\in\boldsymbol{r}} \frac{\varphi_d}{|z_d|} = \sum_{n\in s'_c\cup s'_e} \sum_{j=1}^{|\mathcal{Z}(n)|} \frac{\varphi_{(j)}}{|z_{(j)}|}(2^{|\mathcal{Z}(n)|-j}-1).$$

This gives us the sum of the relation integrated information of all the relations, except the self-relations, i.e. $|\boldsymbol{r}| = 1$. The self-relations can be assessed individually without combinatorial explosion.

Analytical count of the number of relations

We can also count all the causal relations among all the distinctions in $D(\mathcal{T}_e, \mathcal{T}_c, s)$ by generalizing the definition of $\mathcal{Z}(n)$ in (1) to all the subsets $o \subseteq s'_c \cup s'_e$:

$$\mathcal{Z}(o) = \{(z,\varphi) : (z,\varphi) \in \mathcal{Z}(\mathcal{T}_e,\mathcal{T}_c,s), z \supseteq o\}.$$

For each distinction $d \in D(\mathcal{T}_e, \mathcal{T}_e, s)$, there is a corresponding element $\big((z^*_c(d) \cup z^*_e(d), \varphi(d)\big)$ in $\mathcal{Z}(o)$ if $o \subseteq z^*_c(d) \cup z^*_e(d)$. Any subset of $\mathcal{Z}(o)$ of size 2 or larger defines a relation whose overlap contains at least o. The number of such subsets is:

$$2^{|\mathcal{Z}(o)|} - |\mathcal{Z}(o)| - 1.$$

We can count all the relations by applying the inclusion-exclusion principle (from combinatorics) as:

$$\sum_{o\subseteq s'_c\cup s'_e} (-1)^{|o|-1}\left(2^{|\mathcal{Z}(o)|} - |\mathcal{Z}(o)| - 1\right).$$

This is the number of all the causal relations among the causal distinctions in $D(\mathcal{T}_e, \mathcal{T}_c, s)$, except the self-relations. Again, the self-relations can be counted individually without combinatorial explosion.
