# Supplementary material for: Integrated information theory (IIT) 4.0: Formulating the properties of phenomenal existence in physical terms
Source: PLoS Comput Biol. 2023 Oct 17;19(10):e1011465. doi: 10.1371/journal.pcbi.1011465 (PMC10581496; doi:10.1371/journal.pcbi.1011465)
Supplement: S1 Fig — Visual summary of the algorithm for identifying complexes and unfolding cause–effect structures. (PDF) [file pcbi.1011465.s004.pdf]

Integrated information theory (IIT) 4.0: Formulating the properties of phenomenal existence in physical terms

Larissa Albantakis[1¶], Leonardo Barbosa[1,2¶], Graham Findlay[1,3¶], Matteo Grasso[1¶], Andrew M Haun[1¶], William Marshall[1,4¶], William GP Mayner[1,3¶], Alireza Zaeemzadeh[1¶], Melanie Boly[1,5], Bjørn E Juel[1,6], Shuntaro Sasai[1,7], Keiko Fujii[1], Isaac David[1], Jeremiah Hendren[1,8], Jonathan P Lang[1], Giulio Tononi[1*]

1 Department of Psychiatry, University of Wisconsin, Madison, Wisconsin, USA

2 Fralin Biomedical Research Institute at VTC, Virginia Tech, Roanoke, Virginia, USA

3 Neuroscience Training Program, University of Wisconsin, Madison, Wisconsin, USA

4 Department of Mathematics and Statistics, Brock University, St. Catharines, Ontario, Canada

5 Department of Neurology, University of Wisconsin, Madison, Wisconsin, USA

6 Institute of Basic Medical Sciences, University of Oslo, Oslo, Norway

7 Araya Inc., Tokyo, Japan

8 Graduate School Language & Literature, Ludwig Maximilian University of Munich, Munich, Germany

¶These authors contributed equally to this work.

* gtononi@wisc.edu

S1 - IIT Algorithm

Let the physical substrate U be a stochastic system of interacting units $\{U_1, U_2, \ldots, U_n\}$.

Let u be its current state, and $u \to \bar{u}$ an update within state space $\Omega_U = \prod_i \Omega_{U_i}$.

Let $\mathcal{T}_U \equiv p(\bar{u}\,|\mathrm{do}(u)) = \prod_{i=1}^{n} p(\bar{u}_i\,|\mathrm{do}(u))$ be its interventional* transition probability function.

(*) Impose all possible current states uniformly.

For each candidate system $S \subseteq U$ in state s and background units $W = U \setminus S$ in state w:

Compute the effect TPM $\mathcal{T}_e \equiv p_e(\bar{s} \mid s) = p(\bar{s} \mid s, w)$

Compute the cause TPM $\mathcal{T}_c \equiv p_c(s \mid \bar{s}) = \prod_{i=1}^{|S|} \sum_{\bar{w}} p(s_i \mid \bar{s}, \bar{w}) \left(\frac{\sum_{\hat{s}} p(u \mid \hat{s}, \bar{w})}{\sum_{\hat{u}} p(u \mid \hat{u})} \right)$

Compute the unconstrained cause probability

$$p_c(s) = |\Omega_S|^{-1} \sum_{\bar{s} \in \Omega_S} p_c(s \mid \bar{s})$$

Compute the probability over $\bar{s}$ using Bayes' rule

$$\overleftarrow{p_c}(\bar{s} \mid s) = \frac{p_c(s \mid \bar{s}) \cdot |\Omega_S|^{-1}}{p_c(s)}$$

For each candidate cause state $\bar{s}$:

Compute intrinsic cause information

$$\mathrm{ii}_c(s, \bar{s}) = \overleftarrow{p_c}(\bar{s} \mid s) \log \left(\frac{p_c(s \mid \bar{s})}{p_c(s)} \right)$$

Find the maximal cause state

$$s'_c(\mathcal{T}_c, s) = \operatorname*{argmax}_{\bar{s} \in \Omega_S} \mathrm{ii}_c(s, \bar{s})$$

Compute the unconstrained effect probability

$$p_e(\bar{s}) = |\Omega_S|^{-1} \sum_{s \in \Omega_S} p_e(\bar{s} \mid s)$$

For each candidate effect state $\bar{s}$:

Compute intrinsic effect information

$$\mathrm{ii}_e(s, \bar{s}) = p_e(\bar{s} \mid s) \log \left(\frac{p_e(\bar{s} \mid s)}{p_e(\bar{s})} \right)$$

Find the maximal effect state

$$s'_e(\mathcal{T}_e, s) = \operatorname*{argmax}_{\bar{s} \in \Omega_S} \mathrm{ii}_e(s, \bar{s})$$

For each directional system partition θ:

Compute the partitioned transition probability functions $\mathcal{T}_c^\theta, \mathcal{T}_e^\theta$

Compute the integrated cause information

$$\varphi_c(\mathcal{T}_c, s, \theta) = \overleftarrow{p_c}(s'_c \mid s) \left| \log \left(\frac{p_c(s \mid s'_c)}{p_c^\theta(s \mid s'_c)} \right) \right|_+$$

Compute the integrated effect information

$$\varphi_e(\mathcal{T}_e, s, \theta) = p_e(s'_e \mid s) \left| \log \left(\frac{p_e(s'_e \mid s)}{p_e^\theta(s'_e \mid s)} \right) \right|_+$$

Compute the (candidate) system integrated information $\varphi_s(\mathcal{T}_c, \mathcal{T}_e, s, \theta) = \min\{\varphi_c(\mathcal{T}_c, s, \theta), \varphi_e(\mathcal{T}_e, s, \theta)\}$

Find the minimum partition (MIP) $\theta' = \operatorname*{argmin}_{\theta \in \Theta(S)} \frac{\varphi_s(\mathcal{T}_c, \mathcal{T}_e, s, \theta)}{\max_{\mathcal{T}'_c, \mathcal{T}'_e} \varphi_s(\mathcal{T}'_c, \mathcal{T}'_e, s, \theta)}$

Identify system integrated information $\varphi_s(\mathcal{T}_e, \mathcal{T}_c, s) := \varphi_s(\mathcal{T}_e, \mathcal{T}_c, s, \theta')$

Find the first complex $S^* = \operatorname*{argmax}_{S \subseteq U} \varphi_s(\mathcal{T}_e, \mathcal{T}_c, s)$. This is the PSC*

(*) In principle, not only sets of units, but also the grain of units, updates, and states should be considered

Unfold the cause-effect structure of the complex:

For each candidate mechanism $M \subseteq S^*$ in state m:

For each candidate purview $Z_c \subseteq S^*$:

Compute the probability over M, marginalizing out external influences $Y = S^* \setminus Z$

$$\pi_c(m \mid z) = \prod_{i=1}^{|M|} |\Omega_Y|^{-1} \sum_{y \in \Omega_Y} p(m_i \mid z, y)$$

Compute the unconstrained cause probability

$$\pi_c(m; Z) = |\Omega_Z|^{-1} \sum_{z \in \Omega_Z} \pi_c(m \mid z)$$

Compute the probability over Z using Bayes' rule

$$\overleftarrow{\pi_c}(z \mid m) = \frac{\pi_c(m \mid z) \cdot |\Omega_Z|^{-1}}{\pi_c(m; Z)}$$

For each candidate cause purview state z:

Compute the intrinsic cause information

$$\mathrm{ii}_c(m, z) = \overleftarrow{\pi_c}(z \mid m) \log \left(\frac{\pi_c(m \mid z)}{\pi_c(m; Z)} \right)$$

Find the maximal cause state

$$z'_c(m, Z) = \operatorname*{argmax}_{z \in \Omega_Z} \mathrm{ii}_c(m, z)$$

For each disintegrating mechanism partition θ:

Compute the partitioned probability

$$\pi_c^\theta(m \mid z'_c) = \prod_{i=1}^{k} \pi_c(m^{(i)} \mid z_c'^{(i)})$$

Compute the integrated cause information

$$\varphi_c(m, Z, \theta) = \overleftarrow{\pi_c}(z'_c \mid m) \left| \log \left(\frac{\pi_c(m \mid z'_c)}{\pi_c^\theta(m \mid z'_c)} \right) \right|_+$$

Find the minimum partition (MIP)

$$\theta' = \operatorname*{argmin}_{\theta \in \Theta(M,Z)} \frac{\varphi_c(m, Z, \theta)}{\max_{\mathcal{T}'} \varphi_c(m, Z, \theta)}$$

Identify integrated information

$$\varphi_c(m, Z) := \varphi_c(m, Z, \theta')$$

Find the maximally irreducible cause

$$z_c^*(m) = \operatorname*{argmax}_{Z \subseteq S} \varphi_c(m, z'_c(m, Z))$$

Identify the integrated cause information

$$\varphi_c(m) := \max_{Z \subseteq S} \varphi_c(m, z'_c(m, Z))$$

For each candidate purview $Z_e \subseteq S^*$:

Compute the probability over Z, marginalizing out external influences $X = S^* \setminus M$

$$\pi_e(z \mid m) = \prod_{i=1}^{|Z|} |\Omega_X|^{-1} \sum_{x \in \Omega_X} p(z_i \mid m, x)$$

Compute the unconstrained effect probability

$$\pi_e(z; M) = |\Omega_M|^{-1} \sum_{m \in \Omega_M} \pi_e(z \mid m)$$

For each candidate effect purview state z:

Compute the intrinsic effect information

$$\mathrm{ii}_e(m, z) = \pi_e(z \mid m) \log \left(\frac{\pi_e(z \mid m)}{\pi_e(z; M)} \right)$$

Find the maximal effect state

$$z'_e(m, Z) = \operatorname*{argmax}_{z \in \Omega_Z} \mathrm{ii}_e(m, z)$$

For each disintegrating mechanism partition θ:

Compute the partitioned probability

$$\pi_e^\theta(z'_e \mid m) = \prod_{i=1}^{k} \pi_e(z_e'^{(i)} \mid m^{(i)})$$

Compute the integrated effect information

$$\varphi_e(m, Z, \theta) = \pi_e(z'_e \mid m) \left| \log \left(\frac{\pi_e(z'_e \mid m)}{\pi_e^\theta(z'_e \mid m)} \right) \right|_+$$

Find the minimum partition (MIP)

$$\theta' = \operatorname*{argmin}_{\theta \in \Theta(M,Z)} \frac{\varphi_e(m, Z, \theta)}{\max_{\mathcal{T}'} \varphi_e(m, Z, \theta)}$$

Identify integrated information

$$\varphi_e(m, Z) := \varphi_e(m, Z, \theta')$$

Find the maximally irreducible effect

$$z_e^*(m) = \operatorname*{argmax}_{Z \subseteq S} \varphi_e(m, z'_e(m, Z))$$

Identify the integrated effect information

$$\varphi_e(m) := \max_{Z \subseteq S} \varphi_e(m, z'_e(m, Z))$$

Compute the candidate distinction's integrated information $\varphi_d(m) = \min\big(\varphi_c(m), \varphi_e(m)\big)$

The candidate distinction is $d(m) = (m,\ z^* = \{z_c^*, z_e^*\},\ \varphi_d)$

Compute the set of congruent distinctions $D(\mathcal{T}_e, \mathcal{T}_c, s^*) = \{d : \varphi_d > 0,\ z_c^* \subseteq s'_c,\ z_e^* \subseteq s'_e\}$

For each candidate set of distincitons $\boldsymbol{d} \subseteq D(\mathcal{T}_e, \mathcal{T}_c, s^*)$:

For each set of causes and/or effects $\boldsymbol{z}$ such that:

$$\boldsymbol{z} \ :\ \boldsymbol{z} \cap \{z_c^*(d), z_e^*(d)\} \neq \varnothing \ \ \forall d \in \boldsymbol{d},\ \bigcap_{z^* \in \boldsymbol{z}} z^* \neq \varnothing,\ |\boldsymbol{z}| > 1$$

Compute the maximal overlap $o^*(\boldsymbol{z}) = \bigcap_{z \in \boldsymbol{z}} z \neq \varnothing$

The relation face is $f(\boldsymbol{z}) = \big(\boldsymbol{z}, o^*(\boldsymbol{z})\big)$

The set of relation faces is $\boldsymbol{f}(\boldsymbol{d}) = \{f(\boldsymbol{z})\}_{\boldsymbol{d}}$

Compute the integrated information of the candidate relation

$$\varphi_r(\boldsymbol{d}) = \min_{d \in \boldsymbol{d}} \left| \bigcup_{f \in \boldsymbol{f}(\boldsymbol{d})} o_f^* \right| \frac{\varphi_d}{|z_c^*(d) \cup z_e^*(d)|}$$

The candidate relation is $r(\boldsymbol{d}) = \big(\boldsymbol{d}, \boldsymbol{f}(\boldsymbol{d}), \varphi_r\big)$

Compute the set of relations $R(D) = \{r(\boldsymbol{d}) : \varphi_r(\boldsymbol{d}) > 0\}$

The cause-effect structure of the complex S^* (its Φ-structure) is

$$C(\mathcal{T}_e, \mathcal{T}_c, s^*) = D \cup R(D) = \{D(\mathcal{T}_e, \mathcal{T}_c, s^*) \cup R(D(\mathcal{T}_e, \mathcal{T}_c, s^*))\}$$

Compute $\Phi(\mathcal{T}_e, \mathcal{T}_c, s^*) = \sum_{C(\mathcal{T}_e, \mathcal{T}_c, s^*)} \varphi.$

existence
intrinsicality
information
integration
exclusion

composition
existence, intrinsicality
information
integration
exclusion

exclusion
integration
