# Supplementary material for: Integrated information theory (IIT) 4.0: Formulating the properties of phenomenal existence in physical terms
Source: PLoS Comput Biol. 2023 Oct 17;19(10):e1011465. doi: 10.1371/journal.pcbi.1011465 (PMC10581496; doi:10.1371/journal.pcbi.1011465)
Supplement: S1 Notes — (PDF) [file pcbi.1011465.s005.pdf]

Integrated information theory (IIT) 4.0: Formulating the properties of phenomenal existence in physical terms

Larissa Albantakis[1¶], Leonardo Barbosa[1,2¶], Graham Findlay[1,3¶], Matteo Grasso[1¶], Andrew M Haun[1¶], William Marshall[1,4¶], William GP Mayner[1,3¶], Alireza Zaeemzadeh[1¶], Melanie Boly[1,5], Bjørn E Juel[1,6], Shuntaro Sasai[1,7], Keiko Fujii[1], Isaac David[1], Jeremiah Hendren[1,8], Jonathan P Lang[1], Giulio Tononi[1*]

1 Department of Psychiatry, University of Wisconsin, Madison, Wisconsin, USA

2 Fralin Biomedical Research Institute at VTC, Virginia Tech, Roanoke, Virginia, USA

3 Neuroscience Training Program, University of Wisconsin, Madison, Wisconsin, USA

4 Department of Mathematics and Statistics, Brock University, St. Catharines, Ontario, Canada

5 Department of Neurology, University of Wisconsin, Madison, Wisconsin, USA

6 Institute of Basic Medical Sciences, University of Oslo, Oslo, Norway

7 Araya Inc., Tokyo, Japan

8 Graduate School Language & Literature, Ludwig Maximilian University of Munich, Munich, Germany

¶These authors contributed equally to this work.

* gtononi@wisc.edu

S1 - Footnotes

1) A substrate should be understood as a set of units that can be observed and manipulated.

2) As mentioned in the section "Determining maximal unit grains," a substrate unit must be maximally irreducible within, which is likely the case for "real" neurons in the brain, but is not the case for "virtual," simulated neurons in a computer program.

3) Tononi, G. On Being (forthcoming book).

4) Strictly speaking, distinctions and relations that can be singled out phenomenally, such as a spot, a book, and so on, correspond, in physical terms, to bundles of distinctions and relations (compound distinctions and relations)—that is, to sub-structures of a Φ-structure (Φ-folds) [1, 2]. This can be understood in neural terms because attentional mechanisms can only highlight subsets of units, and thereby all the associated distinctions and relations, rather than individual distinctions and relations. In other words, introspection is the starting point for an explanation of experience in physical terms, but it can only go so far. A full explanation can only be provided through a back-and-forth between the properties of a substrate, which can be explored in great detail, and the properties of experience, which can only be characterized crudely through introspection.

5) Whether these assumptions are ultimately compatible with fundamental physics remains to be determined. However, it is only consistent to assume that the TPM should include all causally relevant aspects of a system and causation may still be discrete even if the system's evolution may be described in terms of continuous fields.

6) While the IIT formalism can be applied to hypothetical or simulated systems (as we do for the example systems in the "Results and discussion" section), for the resulting quantities to capture existence in physical terms they must be applied to substrate units that can actually be observed and manipulated in physical terms.

7) As demonstrated in [3], it is possible to extend IIT's causal framework to finite quantum systems under unitary evolution, where the conditional independence assumption applies to non-entangled subsystems.

8) Note that this notion of irreducibility based on set-partitions differs from typical information-theoretic notions such as redundancy or synergy [4–6].

9) A principle of IIT not discussed here is the Principle of becoming, which states that powers become what powers do. That is, conditional probabilities in the TPM update depending on what happens. The principle and some of its implications are examined in (3).

10) “Strongly connected” means that every node can be reached from every other node in a directed graph.

11) Marshall W, Findlay G, Albantakis L, Tononi G. A Mathematical Framework for Cause-Effect Power Analysis of Macro Units (in prep.).

12) Units within the candidate system are causally marginalized based on a uniform distribution to discount their causal contribution if they are not part of the mechanism or purview under consideration. By contrast, units outside the candidate system (background conditions) are causally marginalized conditional on the current state of the universe, potentially leading to a non-uniform distribution.

13) It is useful to note that we can partition a cause-effect structure into distinction $\varPhi$-folds as follows. To do so, we assume that each distinction contributes equally to the existence of a relation $r(\boldsymbol{d})$, because removing any distinction $d \in \boldsymbol{d}$ will “unrelate” the set $\boldsymbol{d}$. Thus, we assign the proportion of $\varphi_r(\boldsymbol{d})$ that each individual distinction $d \in \boldsymbol{d}$ contributes to the full quantity to be $\varphi_r(d) = \varphi_r(\boldsymbol{d})/|\boldsymbol{d}|$. We can then define the $\varPhi_d$ of a distinction $\varPhi$-fold $C(\{d\})$ as the sum of all $\varphi_r(d)$ values of each relation in $C(\{d\})$. The $\varPhi_d$ values of all distinction $\varPhi$-folds with $d \in D$ then sum to the $\varPhi$ value of the entire cause-effect structure $C(D)$.

14) Boly, M et al. Neural correlates of pure presence (in prep.).

15) The result of [7] is being extended to the updated 4.0 framework: Findlay et al. Dissociating Artificial Intelligence from Artificial Consciousness (in prep.).

16) Comolatti, R et al. Why does time feel flowing (in prep.) and Grasso, M et al. How do phenomenal objects bind general concepts with particular features? (in prep.).

17) Mayner WGP, Juen BE, Tononi G. Meaning, perception, and matching: quantifying how the structure of experience matches the environment (in prep.).

18) Zaeemzadeh, A et al. Shannon Information and Integrated Information (in prep.).

References

1. Haun AM, Tononi G. Why Does Space Feel the Way it Does? Towards a Principled Account of Spatial Experience. Entropy. 2019;21(12):1160. doi:10.3390/e21121160.

2. Ellia F, Hendren J, Grasso M, Kozma C, Mindt G, Lang JP, Haun AM, Albantakis L, Boly M, and Tononi G. Consciousness and the fallacy of misplaced objectivity. Neuroscience of Consciousness. 2021;2021(2):1–12. doi:10.1093/NC/NIAB032.

3. Albantakis L, Prentner R, Durham I. Measuring the integrated information of a quantum mechanism. Entropy. 2023;25.

4. Albantakis L, Tononi G. Causal Composition: Structural Differences among Dynamically Equivalent Systems. Entropy 2019, Vol 21, Page 989. 2019;21(10):989. doi:10.3390/E21100989.

5. Beer RD, Williams PL. Information processing and dynamics in minimally cognitive agents. Cognitive Science. 2015;39(1):1–38. doi:10.1111/cogs.12142.

6. Mediano PAM, Rosas F, Carhart-Harris RL, Seth AK, Barrett AB. Beyond integrated information: A taxonomy of information dynamics phenomena. arXiv. 2019;1909.02297.

7. Findlay G, Marshall W, Albantakis L, Mayner WGP, Koch C, Tononi G. Dissociating Intelligence from Consciousness in Artificial Systems – Implications of Integrated Information Theory. In: Proceedings of the 2019 Towards Conscious AI Systems Symposium, AAAI SSS19; 2019.
